# Recombinant Dense Granular Protein (GRA5) for Detection of Human Toxoplasmosis by Western Blot

**DOI:** 10.1155/2014/690529

**Published:** 2014-05-29

**Authors:** Xiao Teng Ching, Yee Ling Lau, Mun Yik Fong, Veeranoot Nissapatorn, Hemah Andiappan

**Affiliations:** Department of Parasitology, Faculty of Medicine, University of Malaya, 50603 Kuala Lumpur, Malaysia

## Abstract

*Toxoplasma gondii* infects all warm-blooded animals, including humans, causing serious public health problems and great economic loss for the food industry. Commonly used serological tests require costly and hazardous preparation of whole *Toxoplasma* lysate antigens from tachyzoites. Here, we have evaluated an alternative method for antigen production, which involved a prokaryotic expression system. Specifically, we expressed *T. gondii* dense granular protein-5 (GRA5) in *Escherichia coli* and isolated it by affinity purification. The serodiagnostic potential of the purified recombinant GRA5 (rGRA5) was tested through Western blot analysis against 212 human patient serum samples. We found that rGRA5 protein was 100% specific for analysis of toxoplasmosis-negative human sera. Also, rGRA5 was able to detect acute and chronic *T. gondii* infections (sensitivities of 46.8% and 61.2%, resp.).

## 1. Introduction

Toxoplasmosis is a parasitic disease caused by* Toxoplasma gondii* (*T. gondii*) which belongs to phylum Apicomplexa [1]. It is an obligate intracellular protozoan parasite capable of infecting all warm-blooded domestic animals as well as human beings [2]. The infection is globally distributed affecting up to one-third of the world's human population [3]. Infection of* T. gondii* involves the transmission within and between hosts by zoites [4]. Three infectious stages of the parasite are tachyzoite, bradyzoite, and sporozoite [5]. Humans get infected with such disease through congenital transmission, consumption of raw or undercooked meat contaminated with* T. gondii* tissue cysts, or uptake of water contaminated with sporulated oocysts from the infected cat feces [6].

Toxoplasmosis in immunocompetent individuals is often asymptomatic [7] but can cause severe clinical outcome to immunocompromised patients leading to multisystem organ failure or even death [2]. Meanwhile, primary infection in pregnant women will likely transmit the parasite to the fetus vertically causing congenital toxoplasmosis and might eventually bring about miscarriage in pregnant women [8, 9]. Besides pregnant women, similar infection does occur in sheep and goats [10], giving rise to similar consequence. Abortions in these animals contribute to great economic loss in livestock industry. Therefore, it is crucial to conduct a rapid, highly accurate, and early diagnostic test for* T. gondii* infected patients/hosts for prevention or early treatment.

In general, there are few methods available for conducting laboratory diagnosis of toxoplasmosis including serologic assays (antibody detection), polymerase chain reaction (PCR; specific gene detection), histologic examination, and isolation of the parasite followed by inoculation into peritoneal cavities of mice (*in vivo*) or tissue cultures (*in vitro*) from biopsy tissue and blood/body fluids, respectively, of the infected patients [11]. However, the most commonly used diagnostic test would be the serological test which relies on the* Toxoplasma* lysate antigens (TLAs) from tachyzoites propagated* in vivo *or* in vitro*. There are several disadvantages pertaining to the usage of antigens originating from tachyzoites: high production cost, time consuming, inconstant quality, contamination with extraparasitic components, and exposure of the staff to the harmful living parasites [12]. To overcome this, recombinant DNA technology plays an important role in producing a larger quantity of recombinant antigenic proteins for serodiagnosis of* T. gondii *infection in a safer manner with lower production cost. Besides, recombinant tachyzoite proteins production either through prokaryotic or eukaryotic systems can reduce the variation of quality, enabling the development of a more specific and standardized serological assay.

Previous studies have reported the potential of various specific antigens of* T. gondii* such as the surface proteins [13, 14], microneme proteins [15], rhoptry proteins [16, 17], and dense granule proteins [18, 19] as seromarkers, either as single or multiantigen for detection of* T. gondii*-specific serum antibodies against acute (recently acquired) or/and chronic (distant past)* Toxoplasma* infection.

Dense granule (GRA) proteins are proteins with high immunogenicity [20]. They are found abundantly in both tachyzoites and bradyzoites [21] and make up most of the circulating antigens in the blood stream of an infected host which can be detected as early as a few hours postinfection (acute phase) [22]. GRA proteins were also found during the chronic stage of* T. gondii* infection [20]. As a result, the immunogenicity and prolonged expression of GRA proteins make them one of the promising candidates for recombinant protein production.

A total of 12 GRA proteins with the molecular weight ranging from 21 to 41 kDa have been identified [4, 21, 23–25]. Diagnostic performance of GRA antigens such as GRA2, GRA6, GRA7, and GRA8 has been investigated via ELISA for discriminating acute from chronic* Toxoplasma* infections [18, 19, 26–28]. Recombinant GRA7 was also shown to detect acute* T. gondii* infection more strongly compared to chronic infection [29]. Meanwhile, in our previous study, sensitivity and specificity of recombinant GRA2 for serodiagnosis of* Toxoplasma*-infected patients' sera have also been evaluated through western blot which is capable of discriminating present from past infection [30]. More diagnostic candidates capable of detecting the early acquired phase of toxoplasmosis ought to be determined to improve the efficacy of serodiagnosis especially of pregnant women in order to reduce the risk of transplacental transmission.

Dense granule antigen 5 (GRA5) is a 21 kDa hydrophobic protein consisting of a N-terminal hydrophobic signal peptide and a hydrophobic transmembrane domain [31]. It was reported that GRA5 appears in both soluble and hydrophobic forms [32]. GRA5 is secreted into the parasitophorous vacuole (PV) by* T. gondii* as a soluble form during the host cell invasion [33] followed by transmembrane insertion into the parasitophorous vacuole membrane (PVM) with its N-terminal projecting into the host cell cytoplasm, while C-terminus remains in the vacuole lumen [32]. A yeast two-hybrid analysis with GRA5 [34] showed that this antigen binds to calcium modulating ligand (CALMG) for regulation of intracellular calcium concentration which helps to inhibit apoptosis [35] and further allows for long-term survival of* T. gondii*. Besides playing an important role in host cell invasion, maintenance of the PV, and long-term survival of the parasite, GRA5 was found to exist in all life stages of the parasite [36].

However, only limited studies were done on the evaluation of the potential of GRA5 as a diagnostic marker in* Toxoplasma* infection, thus making it a protein of interest to be studied in this research. Only one study has been conducted showing the suitability of the full-length recombinant GRA5 for use as a component of an antigen cocktail for the detection of anti-*T. gondii* IgG antibodies [37]. This research study was aimed at the production of recombinant GRA5 (designated rGRA5) antigen in bacteria and at evaluation of its immunogenic properties as a potential single-antigenic diagnostic candidate through western blot. At the same time, we will also find out if GRA5 can detect the early acute stage of human toxoplasmosis through this study.

## 2. Materials and Methods

### 2.1. Parasite

*T. gondii* tachyzoites (RH strain) were maintained by serial intraperitoneal passage in BALB/c mice and were harvested from the peritoneal fluids after 3 to 4 days of infection. The tachyzoites were washed and subsequently resuspended in sterile phosphate buffered saline (PBS) prior to usage.

### 2.2. Construction of Recombinant Plasmids

The* T. gondii GRA5* gene sequence (corresponding to nucleotides 76–360), which encodes the GRA5 antigen, was obtained from Genbank (accession number: EU918733.1). DNA was extracted from tachyzoites of* T. gondii* (RH strain) and used as the template for PCR amplification of the* GRA5* gene with forward (5′-GCGGAATTCGGTTCAACGCGTGAC-3′) and reverse (5′-GACGAATTCCTCTTCCTCGGCAACTTC-3′) primers, which introduced* EcoR*I restriction sites (underlined) to facilitate cloning. The PCR product was purified and cloned into the pRSET B prokaryotic expression vector (Invitrogen, USA) at the EcoRI site. The resulting recombinant GRA5-pRSET B construct permitted expression of an N-terminally polyhistidine- (His-) tagged rGRA5 (amino acid residues 26–120), lacking its putative N-terminal signal sequence. Both the GRA5-pRSET B construct and the nonrecombinant pRSET B plasmid were transformed into the prokaryotic expression host,* Escherichia coli* (*E. coli*) BL21(DE3)pLysS. The recombinant clones were screened and sequenced for verification purposes.

### 2.3. Optimization of Heterologous Protein Expression in* E. coli*

Optimal conditions for rGRA5 protein expression in* E. coli* were determined prior to scaling up the protein production protocol for further study. A single GRA5-pRSET B-containing colony was picked and inoculated into 5 mL of Luria-Bertani (LB) broth supplemented with ampicillin (100 *μ*g/mL) and chloramphenicol (34 *μ*g/mL). The culture was grown overnight at 37°C (200 rpm) and then diluted to a final volume of 10 mL with LB broth to yield an optical density of 0.1 at 600 nm (OD_600_). The culture was then grown at 37°C (~250 rpm) until reaching an OD_600_ of 0.5, at which point protein expression was induced by addition of different concentrations (0.1, 0.5, and 1.0 mM) of isopropyl *β*-D-thiogalactopyranoside (IPTG; Invitrogen, USA) for various incubation periods (0, 2, and 4 h). The cells were harvested every hour by centrifugation at 5,000 ×g for 10 min before assessing protein expression using dodecyl sulphate-polyacrylamide gel electrophoresis (SDS-PAGE).

### 2.4. Expression and Purification of rGRA5

Large-scale protein production was achieved by inducing the culture with 1 mM IPTG and incubating it for 2 h before harvesting by centrifugation. The Probond Purification System (Invitrogen, USA) and nitrilotriacetic acid-nickel (Ni-NTA; Qiagen, Germany) resin were then used to purify rGRA5, according to the manufacturers' instructions. Briefly, cell lysate was prepared under denaturing conditions prior to the purification steps. The cell pellet was resuspended in guanidine lysis buffer (6 M guanidine hydrochloride, 500 mM sodium chloride, and 20 mM sodium phosphate, pH 7.8) and rocked slowly for 5 to 10 min at room temperature to ensure thorough cell lysis, followed by sonication on ice with three 5-second pulses (high intensity). After sonication, the lysate was separated from cellular debris by centrifugation at 3,000 ×g for 15 min, added to a column with resin, and allowed to bind for 30 min. Once the resin settled, the supernatant was aspirated, and the column was washed two times with each of the following: denaturing binding buffer (8 M urea, 500 mM sodium chloride, and 20 mM sodium phosphate, pH 7.8), denaturing wash buffer (8 M urea, 500 mM sodium chloride, and 20 mM sodium phosphate, pH 6.0), and denaturing wash buffer (8 M urea, 500 mM sodium chloride, and 20 mM sodium phosphate, pH 5.3). The supernatant was aspirated after each washing step. After the last wash, the rGRA5 protein was eluted from the Ni-NTA resin with denaturing elution buffer (8 M urea, 500 mM sodium chloride, and 20 mM sodium phosphate, pH 4.0).* E. coli *carrying the empty pRSET B vector was used as a negative control for both expression and purification. The concentration of purified rGRA5 protein was measured with the Bradford Assay Kit (Bio-Rad, USA). The identity of the expressed and purified rGRA5 protein was confirmed by matrix-assisted laser desorption/ionization-time-of-flight (MALDI-TOF) mass spectrometry (MS).

### 2.5. In-Gel Tryptic Digestion of rGRA5

Affinity purified rGRA5 was resolved by SDS-PAGE using 12% polyacrylamide gels, which were stained with Coomassie Brilliant Blue R-250 (Bio-Rad, USA) for 2 h and then incubated with destaining solution (7% acetic acid, 5% methanol) overnight at room temperature. The rGRA5 protein band was then excised from the Coomassie-stained gel (based on size) and further destained with 50 *μ*L of 50% acetonitrile (ACN) in 50 mM ammonium bicarbonate (NH_4_HCO_3_). This step was repeated several times (15–20 min washes, discarding the destaining solution after each wash) until the excised gel was completely destained. The rGRA5-containing gel plug was then incubated with 150 *μ*L of 10 mM dithiothreitol (DTT) in 100 mM NH_4_HCO_3_ for 30 min at 60°C. The gel was subsequently cooled to room temperature, the DTT solution was discarded, and the band was incubated with 150 *μ*L of 55 mM iodoacetamide (IAA) in 100 mM NH_4_HCO_3_ in the dark for 20 min. The gel plug was then washed four times with 50% ACN in 50 mM NH_4_HCO_3_ (500 *μ*L washes, 20 min each), dehydrated via incubation with 50 *μ*L of 100% ACN for 15 min, and subjected to speed vacuum for 15 min at ambient temperature to remove the ACN. The gel plug was then incubated with 25 *μ*L of trypsin (6 ng/*μ*L) in 50 mM NH_4_HCO_3_ at 37°C. Following overnight digestion, 50 *μ*L of 50% ACN was added to the gel plug, and it was incubated for 15 min in order to disintegrate the trypsin enzyme and extract protein from the gel plug. The resulting liquid (containing the digested protein) was transferred into a new tube (Tube A), and the gel plug, which remained in the old tube, was further incubated with 50 *μ*L of 100% ACN for 15 min. Subsequently, this liquid was also transferred to Tube A. The protein-containing solution in Tube A was then dried completely via speed vacuum. Prior to MALDI-TOF MS analysis, the protein sample was reconstituted in 10 *μ*L of 0.1% formic acid and desalted using a Zip-Tip (Millipore, USA). For this, the Zip-Tip membrane was wetted and equilibrated with 50% ACN and 0.1% formic acid, respectively. The protein sample was bound onto the Zip-Tip membrane, which was then washed with 0.1% formic acid. Finally, the protein was eluted with 0.1% formic acid in 50% ACN and analyzed by MALDI-TOF MS.

### 2.6. MALDI-TOF MS Analysis

The Zip-Tip-eluted protein sample was mixed at a 1 : 1 ratio. The matrix was provided by UMCPR staff before spotting onto the MALDI plate. The analysis was carried out by University Malaya Center for Proteomics Research (UMCPR).

### 2.7. SDS-PAGE and Western Blot Analysis

Purified rGRA5 protein was resolved by SDS-PAGE on 12% polyacrylamide gels and transferred onto methanol-activated polyvinylidene difluoride (PVDF; Bio-Rad, USA) membranes, which were then cut into vertical strips. The membranes were incubated with blocking solution (5% nonfat skim milk in Tris Buffered Saline (TBS)) for 2 h at room temperature with constant shaking and were subsequently probed with diluted human serum samples (1 : 200) for 2 h. The membrane strips were washed and then incubated for 1 h with biotinylated goat anti-human IgM/IgG (KPL, USA; 1 : 2500) secondary antibody. Lastly, the membrane strips were washed and incubated with streptavidin-alkaline phosphatase (KPL, USA; 1 : 2,500) at room temperature for 1 h followed by detection using 5-bromo-4-chloro-3-indolyphosphate/nitro blue tetrazolium (BCIP/NBT; Sigma, USA).

### 2.8. Evaluation of Sensitivity and Specificity of rGRA5

Diagnostic sensitivity and specificity of rGRA5 protein were evaluated by western blot analysis using sera from both toxoplasmosis-diagnosed patients and toxoplasmosis-negative individuals. Toxoplasmosis cases were divided into three groups: (1) patients with early acute toxoplasmosis (*n* = 44; IgM positive, IgG negative); (2) patients with acute toxoplasmosis (*n* = 47; IgM positive, IgG positive); and (3) patients with chronic toxoplasmosis (*n* = 85; IgM negative, IgG positive). A fourth group was comprised of toxoplasmosis-negative control patients (*n* = 24; IgM negative, IgG negative). These human serum samples were grouped based on results obtained from Novalisa* Toxoplasma gondii* IgG and* Toxoplasma gondii* IgM enzyme-linked immunosorbent assay (ELISA) kits (NovaTec, Germany). In addition, specificity of rGRA5 was determined using serum samples from patients diagnosed with amoebiasis (3 samples), cysticercosis (3 samples), filariasis (3 samples), and toxocariasis (3 samples). These sera had given positive results in serological tests for their respective infections. All serum samples were obtained from the Diagnostic Laboratory at the Department of Parasitology, University of Malaya. Sensitivity (number of true positives/[number of true positives + number of false negatives]) and specificity (number of true negatives/[number of true negatives + number of false positives]) were calculated and tabulated in Table 1.

## 3. Results

### 3.1. Cloning of the* GRA5* Gene Fragment

We PCR-amplified a fragment of* T. gondii GRA5* gene, which encoded amino acids 26–120 of the GRA5 protein (excluding the putative hydrophobic signal peptide). The resulting ~285 bp product was cloned into the pRSET B vector in order to permit prokaryotic expression of N-terminally His-tagged rGRA5, which could be purified using a nickel resin column. Sequence analysis confirmed that the insert within the GRA5-pRSET B plasmid shared 100% identity with the published* GRA5* gene.

### 3.2. Optimization of rGRA5 Expression in* E. coli*

**Figure 1:**
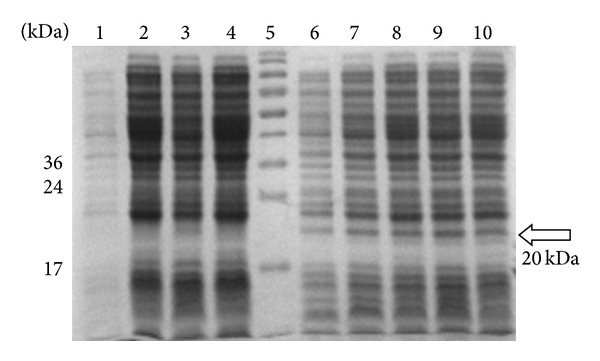
SDS-PAGE analysis on the optimized expression of rGRA5 protein in* E. coli* BL21 pLysS (DE3), Coomassie blue stained. Lane 5: prestained broad range protein marker. Lane 1, cell pellet fractions of pRSET B clone as negative control before induction (0 hr). Lane 2: cell pellet fractions of pRSET B clone after induction with 0.5 mM IPTG (4 hr). Lanes 3 to 4: cell pellet fractions of pRSET B clone after induction with 1.0 mM IPTG (2, 4 hr). Lane 6: cell pellet fractions of GRA5 clone before induction (0 hr). Lanes 7 to 8: cell pellet fractions of GRA5 clone after induction with 0.5 mM IPTG (2, 4 hr). Lanes 9 to 10: cell pellet fractions of GRA5 clone after induction with 1.0 mM IPTG (2, 4 hr). The GRA5 protein band of interest was observed at molecular weight of 20 kDa (arrow) compared to the negative control. The band intensity increased from 0 to 2 hr after induction and remained constant at the 4th hr with 1.0 mM IPTG, the optimum condition for maximum expression of the protein.

Production of rGRA5 protein was optimized by altering various parameters, and expression levels were analyzed by SDS-PAGE as shown in Figure 1. Upon induction of rGRA5 expression from GRA5-pRSET B-containing* E. coli*, we observed a 20 kDa band of increasing intensity, which was absent in the negative control (empty pRSET B). Expression of this protein increased up to two hours after induction and remained constant after four hours. Three different IPTG concentrations were tested, and 1.0 mM was found to result in maximum rGRA5 expression. Taken together, these data suggested that optimal rGRA5 expression was achieved following induction with 1.0 mM IPTG for 2 hours. The same conditions were applied to larger scale production of rGRA5.

### 3.3. Expression and Purification of rGRA5 Protein

**Figure 2:**
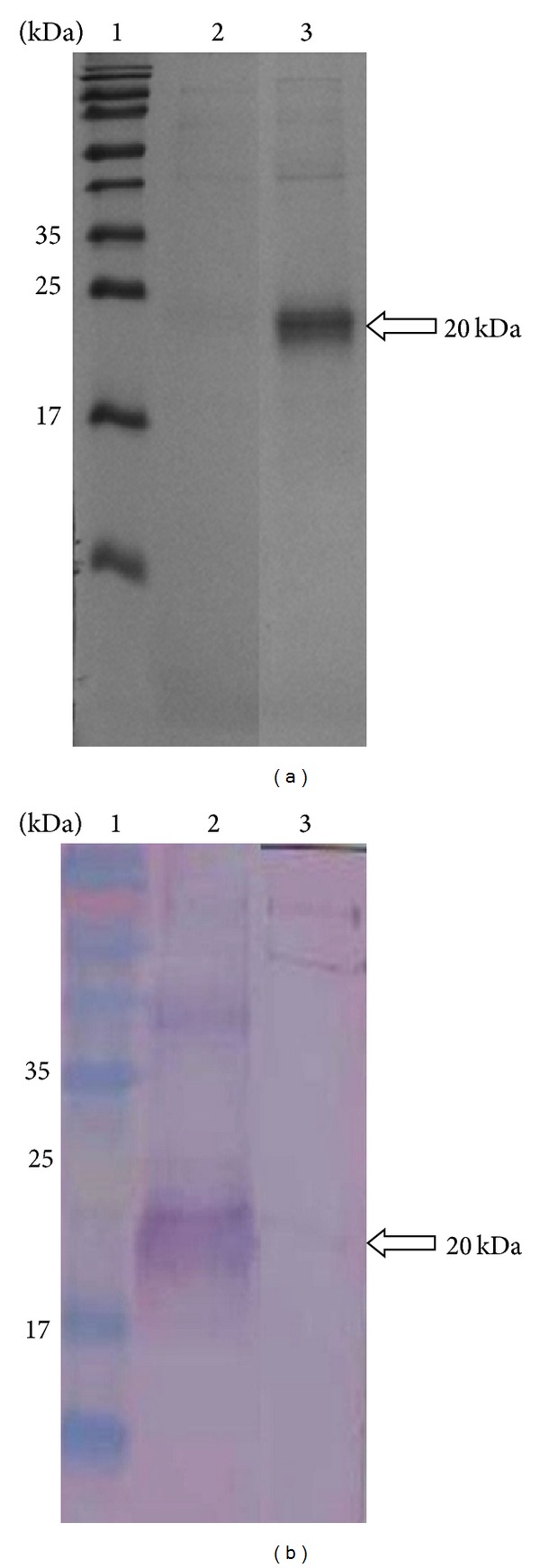
SDS-PAGE analysis on purified rGRA5 protein. (a) Coomassie blue stained. Lane 2: purified pRSET B. Lane 3: purified rGRA5 and (b) western blot probed with toxoplasmosis-infected patient's serum. Lane 2: purified rGRA5. Lane 3: purified pRSET B. Lane 1 (panel a and panel b) is the prestained broad range protein marker. The 20 kDa purified rGRA5 was detected (arrow).

Following optimization of rGRA5 expression in* E. coli*, a nickel resin column was used to purify the recombinant protein (Figure 2(a)), which could be detected by western blot analysis using serum from a* Toxoplasma*-infected patient (Figure 2(b)). This further suggested that the induced 20 kDa band observed prior to purification corresponded to rGRA5 (Figure 1).

### 3.4. Confirmation of rGRA5 Protein

Next, we confirmed the identity of our expressed and purified recombinant protein by MALDI-TOF MS analysis. Indeed, the results indicated that the isolated protein was* T. gondii* GRA5.

### 3.5. Western Blot Analysis of rGRA5 Protein with Human Serum Samples

**Figure 3:**
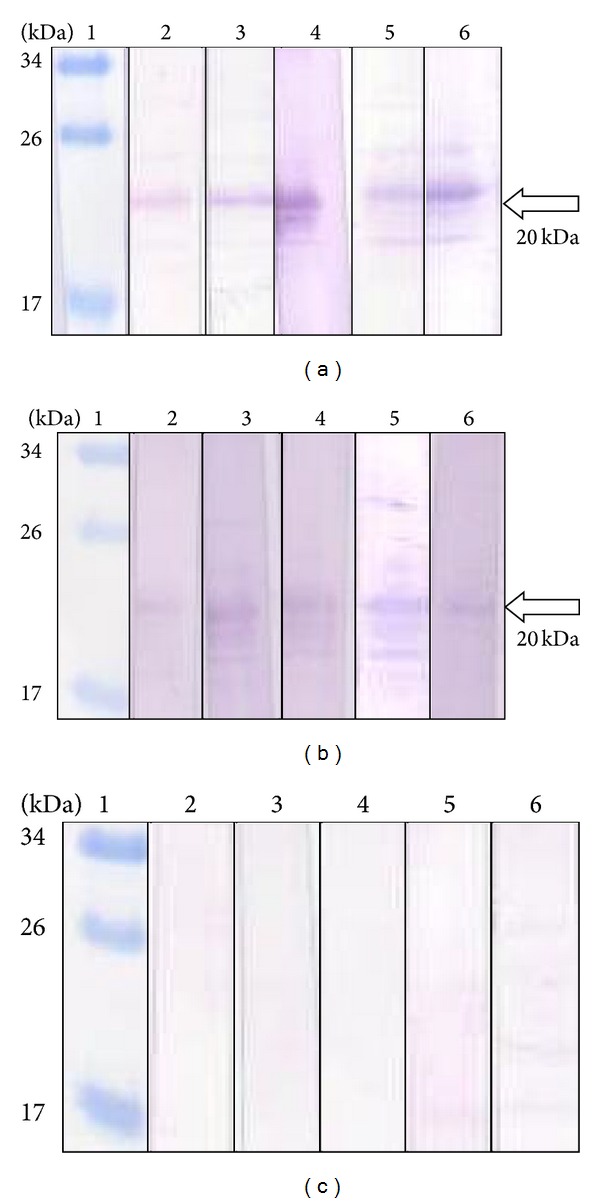
Western blots of purified rGRA5 protein with sera of toxoplasmosis and toxoplasmosis-negative patients. Lane 1 (panels a–c): the prestained broad range protein marker. Lanes 2 (a) to 6 (a): results of 5 sera from chronic-profile patients (Group 3: IgG +ve, IgM −ve). Lanes 2 (b) to 6 (b): results of 5 sera from acute-profile patients (Group 2: IgG +ve, IgM +ve). Lanes 2 (c) to 6 (c): results of 5 sera from toxoplasmosis-negative patients (Group 4: IgG −ve, IgM −ve). The 20 kDa purified rGRA5 was detected by toxoplasmosis-positive sera (arrow).

The purified rGRA5 protein was tested for its diagnostic sensitivity and specificity through western blot analysis with serum samples from toxoplasmosis-positive (Groups 1, 2, and 3) and toxoplasmosis-negative (Group 4) patients. In addition, specificity was tested using sera from patients infected with other parasites, including amoebiasis, cysticercosis, filariasis, and toxocariasis. We observed that the rGRA5 protein had sensitivities of 0% (0 out of 44 sera), 46.8% (22 out of 47 sera), and 61.2% (52 out of 85 sera) for early acute, acute, and chronic infections, respectively (Table 1). In contrast, 0 out of 24 control sera from the toxoplasmosis-negative patients reacted with rGRA5 (100% specificity). In Figure 3, five example results are shown for each group (positive results for Groups 2 and 3; negative results for Group 4). Also, only 1 (toxocariasis) out of the 12 sera from patients infected with other parasites (data not shown) reacted with the rGRA5 protein (91.7% specificity).

## 4. Discussion

A fragment of the* T. gondii GRA5* gene was successfully cloned into a prokaryotic expression vector and transformed into* E. coli*. Full-length recombinant GRA5 protein (rGRA5) was subsequently expressed and purified, yielding a 20 kDa protein. However, the predicted molecular weight of GRA5 is 16 kDa. While this discrepancy between the calculated and observed molecular weights can be partially explained by the presence of the His-tag in rGRA5, it is also possible that this difference stems from common features of GRA proteins, such as proline residue composition [4]. Even though we observed this size discrepancy, the identity of our purified protein was verified by immunoblotting with* Toxoplasma*-infected sera and MALDI-TOF MS analysis.

Identification of rGRA5 via MALDI-TOF MS involved careful processing, which allowed for reliable confirmation of the purified protein. Briefly, the rGRA5-containing band was excised from a stained SDS-PAGE gel, followed by an in-gel digestion protocol that included seven major steps: (1) destaining of the gel plug, (2) reduction of the protein, (3) alkylation of the protein, (4) dehydration, (5) tryptic digestion of the protein, (6) extraction of the digested protein, and (7) desalting of the digested protein using a Zip-Tip. Reduction and alkylation (aminocarboxymethylation) of the protein at cysteine residues with dithiothreitol (DTT) and iodoacetamide (IAA), respectively, were important for permanent disruption of disulfide linkages, enabling overnight trypsin digestion. 

It was demonstrated that the expression of predicted immunodominant epitopes of GRA5 failed to show any immunoreactivity with a pool of* T. gondii*-positive human sera [13]. Therefore, full-length rGRA5 was constructed and produced in this study. Our evaluation of rGRA5 immunoreactivity revealed high specificities when testing sera from toxoplasmosis-negative patients or from those infected with other parasites (100.0% and 91.7%, resp.). In addition, our findings indicate the sensitivities of 46.8% and 61.2% when analyzing serum samples from patients with acute and chronic* Toxoplasma* infections, respectively. However, none of the serum samples from the early acute phase patients reacted with the rGRA5 protein. In fact, data from the present study are in agreement with previous results obtained from analysis of rGRA5 antigen-mediated detection of IgG antibodies using ELISA [37]. Specificity of the aforementioned study was shown to be 100.0%, whereas sensitivities of 63.0% and 75.0% were reported for sera from acute and chronic infections, respectively. Thus, it strongly suggested that rGRA5 yields a much higher reactivity towards IgG antibodies in sera from chronically infected patients compared to those with acute infection. Notably, this protein shows no sensitivity towards IgM antibodies found in sera from early acute stage patients. Our study involved the same expression host, BL21(DE3)pLysS, for the expression of full-length rGRA5 as the above mentioned study. In contrast, different expression vectors and evaluation techniques were used. Due to its higher specificity, western blot was chosen to evaluate rGRA5 protein in this study instead of the commonly used ELISA. Also, the chances of obtaining false-positive results via western blot are much lower compared to ELISA [38]. In fact, it has been reported that western blot analysis is superior to ELISA for screening sera samples because this technique gives more information, is less affected by sample degradation, produces results of high confidence with direct visualization of antibodies bound to specific diagnostic antigens, and offers improved determination of diagnostic antigen purity [39].

With regard to the future development of diagnostic tests for* T. gondii*, the western blot results obtained in this study should be reliable for predicting the efficacy of using rGRA5 antigen in immunochromatographic tests (ICT) due to similarities between the two assays (i.e., western blot and ICT are both immunoassays utilizing nitrocellulose membranes and direct visualization of results). Indeed, ICT is a better serological test for diagnosis of infections (including toxoplasmosis) compared to ELISA, which is commonly used due to its simplicity. However, ICT is a rapid test with high accuracy but lower cost compared to ELISA, which is time consuming and laborious [40]. In addition, ICT can be used in field conditions [40] especially for the diagnosis of farm animals.

Based on our results (Table 1), cross-reactivity was not observed in sera samples from patients infected with amoebiasis, cysticercosis, and filariasis. However, one out of three toxocariasis-positive sera samples reacted with the rGRA5 antigen. This particular toxocariasis-positive serum sample was shown to be IgG positive but IgM negative for toxoplasmosis based on findings from Novalisa* Toxoplasma gondii* IgG and IgM antibodies ELISA kits. This indicates that there was probably a coinfection of* T. gondii* and* Toxocara* spp. in this infected patient [41]. Although* T. gondii* (a protozoan) and* Toxocara* spp. (helminths) are two different parasites, they both can be acquired through soil ingestion. Therefore, the chances of coinfection between these two parasites are highly possible [41].

## 5. Conclusions

Our findings show that rGRA5 lacks sensitivity for detecting IgM antibodies and displays a much lower reactivity towards IgG antibodies in sera from patients with acute infection compared to those with chronic toxoplasmosis (46.8% versus 61.2%). These data indicate that rGRA5 protein is unable to distinguish between current and past infections. Nevertheless, this protein can be combined with other* T. gondii* antigens (cocktails) in order to improve its sensitivity against toxoplasmosis-positive serum samples [37]. Last but not least, these findings should contribute to the future development of an ICT incorporating this antigen (either alone or in combination with other potential ESA) for diagnosis of* T. gondii* infection.

## Figures and Tables

**Table 1:** Immunoreactivities (sensitivity and specificity) of the rGRA5 antigen to serum samples from toxoplasmosis-positive and toxoplasmosis-negative patients.

Serum samples group	Number of human serum samples	Immunoreactivities			
		Positive		Negative	
		Number	%	Number	%
1 (Early acute: IgG−ve, IgM+ve)	44	0	0	44	100
2 (Acute: IgG+ve, IgM+ve)	47	22	46.8	25	53.2
3 (Chronic: IgG+ve, IgM−ve)	85	52	61.2	33	38.8
4 (Toxoplasmosis-negative: IgG−ve, IgM−ve)	24	0	0	24	100
Other infections	12	1	8.3	11	91.7
Amoebiasis	3	0	0	3	100
Cysticercosis	3	0	0	3	100
Filariasis	3	0	0	3	100
Toxocariasis	3	1*	33.3	2	66.7

*One out of three toxocariasis-positive sera samples reacted with the rGRA5 antigen. This particular toxocariasis-positive serum sample was shown to be IgG positive for toxoplasmosis based on the commercial kits.
